# Prevalence of Occult Hepatitis B Virus Infection in Hemodialysis Patients From Egypt With or Without Hepatitis C Virus Infection

**DOI:** 10.5812/hepatmon.665

**Published:** 2012-04-30

**Authors:** Mona A. Abu El Makarem, Mohammed Abdel Hamid, Ashraf Abdel Aleem, Ahmed Ali, Mohammed Shatat, Douaa Sayed, Ali Deaf, Lamia Hamdy, Effat A. Tony

**Affiliations:** 1 Department of Internal Medicine, Medical school, Minia University, Minia, Egypt; 2 Department of Microbiology, Medical school, Minia University, Minia, Egypt; 3 Flow Cytometry Lab, Clinical Pathology Department, South Egypt Cancer Institute, Assiut University, Assiut, Egypt; 4 Department of clinical pathology, Medical school, Minia University, Minia, Egypt; 5 Department of Internal Medicine and Nephrology, Assuit University, Asyut, Minia, Egypt

**Keywords:** Prevalence, Hepatitis B, Hepatitis C, Hemodialysis, Egypt

## Abstract

**Background:**

While prevalence of Hepatitis B virus (HBV) in patients with end-stage renal failure (ESRF) who are undergoing dialysis has decreased significantly during the past few decades, it still remains a distinct clinical problem. The immunosuppressive nature of renal disease often leads to chronicity of the HBV infection and an opportunity for nosocomial spread of the infection among dialysis patients. Egypt is among the countries with intermediate endemicity of HBsAg (range, 2%–7%). Large-scale geographic heterogeneity in HBV prevalence has been reported worldwide and HBV prevalence is especially heterogeneous in Egypt.

**Objectives:**

To assess the prevalence of occult HBV infection (OBI) in hemodialysis patients with or without chronic hepatitis C (HCV) from Minia and Assuit, Upper Egypt, using HBV DNA assays.

**Patient and Methods:**

Sera from 145 hemodialysis patients with negative HbsAg were investigated for HBV DNA using real-time polymerase chain reaction (RT-PCR). Only serum samples with repeatedly detectable HBV DNA were considered positive. Patients were divided into 2 groups: HCV RNA positive and HCV RNA negative, based on the results of a third generation enzyme linked immunosorbent assay (ELISA) anti-HCV test and HCV RNA PCR.

**Results:**

HBV DNA was detected in 6 of the 145 patients (4.1%) and HBcAb was detected in 29/145 patients (20%). There were no statistically significant differences in the age, duration of hemodialysis, biochemical parameters, serological markers of HBV, or HBV DNA between patients with and without HCV infection.

**Conclusions:**

Four percent of the hemodialysis patients had OBI. There was no significant difference in the prevalence of OBI between hemodialysis patients with or without HCV co-infection.

## 1. Background

Infections with hepatitis B virus (HBV) and hepatitis C virus (HCV) are well-known and important causes of liver disease in end-stage renal failure (ESRF) patients on hemodialysis (HD) [1][2][3][4]. Although the prevalence of HBV in patients with ESRF undergoing dialysis has decreased significantly during the past few decades [1], it is still a distinct clinical problem, as the immunosuppressive nature of renal disease often leads to chronicity of the viral infection and results in an opportunity for nosocomial spread of the infection among dialysis patients [1][2][3][4][5]. Occult HBV infection (OBI) is defined as the presence of a small amount of HBV in patients with serum that tests negative for hepatitis B surface antigen (HbsAg). The serum HBV DNA level in these patients is generally lower than 104 copies/mL [6]. There is a high prevalence of OBI in patients with chronic hepatitis C, HCC cryptogenic liver disease, and HIV. OBI is also highly prevalent among hemodialysis patients, those undergoing frequent blood transfusions (as treatment for hemophilia disease, etc.), blood donors, and intravenous drug users [7][8][9][10][11][12][13].

There is intermediate endemicity of HBsAg in Egypt (range, 2%–7%; [14][15][16]. Large-scale geographic heterogeneity in HBV prevalence has been reported worldwide, and prevalence is especially heterogeneous across Egypt [16]. Despite the potential clinical importance of OBI, existing information regarding the prevalence of OBI among Egyptian patients undergoing long-term HD is limited.

## 2. Objectives

We conducted this study at the 2 main referral centers in Upper Egypt (Minia and Assuit) to investigate the prevalence of OBI in HD patients. We used a highly sensitive and specific PCR method to test for OBI and compared OBI prevalence in HCV-positive and HCV negative HD patients. We also correlated the clinical, epidemiologic, and laboratory test characteristics of patients with OBI and with or without HCV co-infection.

## 3. Patient and Methods

A total of 145 patients with end-stage renal disease undergoing regular HD (for at least 6 months) with or without HCV RNA positivity by PCR were included in this study. Patients were enrolled from 2 of the largest nephrology and dialysis units in Upper Egypt (the southern part of Egypt); the dialysis unit of the Department of Internal Medicine, Minia University and the dialysis unit of the Department of Internal Medicine, Assuit University. We defined chronic HCV infection as the presence of anti-HCV antibodies for more than 6 months and positive serum HCV RNA [17]. We excluded patients with acute or chronic HBV infection (as determined by positive HBsAg), were vaccinated against HBV, other causes of liver dysfunction (i.e., primary biliary cirrhosis, autoimmune hepatitis, continued alcohol abuse, autoimmune hepatitis, and HIV infection), or were being treated with interferon and/or ribavirin. We obtained complete medical history for each patient, including age, location of residence, HBV vaccination history, blood transfusion history, duration of hemodialysis, etiology of end-stage renal disease, and history of schistosomiasis. All patients also underwent a complete physical examination. Serum samples were collected between May and August 2009 (before hemodialysis) and then stored at -80C° until tested. Serological markers of HBV infection (HBsAg, anti-HBs, anti-HBc, HBeAg, and anti-HBe) were determined using standard third generation commercially available enzyme immunoassays (Abbott GmbH, Wiesbaden-Delkenheim, Germany).

### 3.1. Detection of HBV DNA by Polymerase Chain Reaction

Samples from each patient were tested for HBV DNA using nested PCR (core fragment) techniques, as described previously [18]. The method uses 4 primers from the X region of the HBV genome and has a lower limit of detection 100 copies/mL. Every sample with HBV DNA detected by nested PCR was tested again using a commercially available real time PCR kit (COBAS Taqman HBV Test; cutoff of detection: 6 IU/mL). The COBAS TaqMan HBV Test is an in vitro nucleic acid amplification test for quantitation of HBV in human serum or plasma, using the High Pure Viral Nucleic Acid kit for manual specimen preparation and the COBAS TaqMan 48 Analyzer for automated amplification and detection. The highly conserved HBV precore/core region is amplified for this test. Only serum samples with repeatedly detectable HBV DNA were considered positive for HBV DNA.

### 3.2. Testing for HCV

Anti-HCV antibody was tested by a third generation enzyme-linked immunosorbent assay (ELISA; HCV 3.0 ELISA Ortho, Raritan, NJ) according to the manufacturer’s instructions. HCV RNA was detected using a qualitative PCR assay (COBAS Amplicor HCV Test, v2.0; Roche Diagnostics; lower limit of diction; 50 IU/mL). Levels of aspartate aminotransferase (AST), alanine aminotransferase (ALT), serum albumin, and bilirubin were determined using standard techniques. Schistosomal antibodies were assessed using an indirect hemagglutination assay with adult Schistosoma mansoni worm antigens (Fumouze Laboratories, Levallois-Perret, France) [19]. The study protocol was approved by the Institutional Ethics Committee at each participating center, and all patients gave informed consent prior to participating in this study. The study was conducted in accordance with the ethical guidelines of the 1975 Declaration of Helsinki and International Conference on Harmonization Guidelines for Good Clinical Practice.

### 3.3. Statistical Analysis

Data were analyzed using Sigma Plot software (SPSS, version 2). We used Chi-square tests (χ(2)) to compare qualitative variables between groups and Student’s two-tailed t-test to compare quantitative variables between groups. Data are presented as percentages, means, and standard deviation (SD). P values lower than 0.05 were considered significant.

## 4. Results

The baseline demographic characteristics of the patients are summarized in Table 1. Patients were 47.4 ± 10.4 years of age (range: 18–69 years), and 82 (56.5%) of the patients were male. Patients were grouped based on HCV PCR status. Group 1 was comprised of 79 patients who were HCV RNA-positive. Group 2 consisted of 66 patients who were HCV RNA-negative. The mean age of the patients in groups 1 and 2 was 48.1 ± 10.5 and 46.5 ± 9.8 years, respectively. In Group 1, 53.1% of the patients were male, while 60.6% of Group 2 patients were male. The mean hemodialysis duration of groups 1 and 2 were 33.2 ± 11 and 31.7 ± 10.07 months, respectively. Seventy (88.6%) and 53 (83.3%) patients in groups 1 and 2, respectively reported a history of past blood transfusion (Table 1). We found no statistically significant differences between the 2 groups based on age, hemodialysis duration or biochemical parameters, except for serum ALT which was significantly higher in group 1 than in group 2 (P = 0.03; Table 1 and 2).

**Table 1:** Clinical and Laboratory Characteristics of Hemodialysis Patients Who Were Positive for (Group 1) and Negative for (Group 2) Hepatitis C Virus RNA

	**All Patients (n = 145)**	**Group 1 (n = 79)**	**Group 2 (n = 66)**	**P value**
Age, y, mean ± SD a	47.4 ± 10.4	48.1 ± 10.5	46.5 ± 9.8	0.3
Sex, No. (%)				0.4
Male	82 (56.5)	42 (53.1)	40 (60.6)	
Female	63 (43.4)	37 (46.8)	26 (39.3)	
Residence, No. (%)				0.4
Rural	110 (75.8)	63 (79.7)	48 (72.7)	
Urban	35 (24.13)	16 (20.2)	18 (27.2)	
Schistosoma antibodies, No. (%)				0.4
Positive	87 (60)	51 (64.5)	36 (54.5)	
Negative	58 (40)	28 (35.4)	30 (45.4)	
Duration of hemodialysis, mo, mean ± SD	33.5 ± 10.6	33.2 ± 11	31.7 ± 10.07	0.1
Positive history of transfusion, No. (%)	125 (86.2)/20 (13.7)	70/79 (88.6)	55/66 (83.3)	0.4
AST a , IU/L, mean ± SD	32.7 ± 4.13	32.03 ± 4.28	31.12 ± 2.97	0.1
ALT a , IU/L, mean ± SD	28.63 ± 5.36	29.81 ± 7.28	27.65 ± 3.65	0.03
Albumin, g/dL, mean ± SD	3.4 ± 0.5	3.42 ± 0.4	3.45 ± 0.5	0.4
Bilirubin, mean ± SD	1.9 ± 0.7	1.83 ± 0.6	1.8 ± 0.5	0.1

^a^ Abbreviations: ALT, alanine transaminase; AST, aspartate transaminase; SD, standard deviation

HBV DNA was detected in 6 of the 145 patients with ESRF (4.1%; 95% CI: 2%–6.1%). When these data were stratified according HCV RNA positivity; HBV DNA was detected through the high-sensitivity PCR study in 4 (5%) and 2 (3%) of group 1 and group 2 patients, respectively (P = 0.2). These 6 patients were considered to have OBI (Table 2). A total of 29 (20%) patients tested positive for HBcAb, with significantly higher ratio in Group 1 (20 patients (25.3%) than in Group 2 patients (29 patients (13.6%)); (P = 0.03) (Table 2). There was no significant correlation between the presence of HBcAb and age, gender, serum AST, ALT, serum albumin, or serum bilirubin. However, in both groups there was a significant relationship between HBcAb and long duration of hemodialysis, history of blood transfusion, and presence of schistosomal antibodies (Table 3). The presence of anti-HBc, in the absence of HBV DNA, suggests recent recovery from an acute HBV.

**Table 2:** Prevalence of HBV Serological Markers as Well as HBV DNA in the Study Population a

	**All Patients (n = 145)**	**Group 1 (n = 79)**	**Group 2 (n = 66)**	**P value**
Anti-HBc b IgG seropositivity	29 (20)	20 (25.3)	9 (13.6)	0.03
Anti-HBs and anti-HBc IgG seropositivity	15 (10.34)	10 (12.6)	5 (7.5)	0.4
Anti-HBc (+), anti-HBe	14 (9.6)	10 (12.6)	4 (6.06)	0.5
HBV DNA c positivity	6 (4.1)	4 (5.06)	2 (3.03)	0.2

^a^ Data are Shown as No. (%).

^b^ Abbreviations: Anti-HBc, hepatitis B core antibody

^c^ HBV DNA, hepatitis B virus DNA by polymerase chain reaction

**Table 3:** Relationship Between Hepatitis B Core Antibody and Clinical and Laboratory Parameters in Hemodialysis Patients With (Group 1) or Without (Group 2) Hepatitis C Virus Infection

	**Group 1 (n = 79)**			**Group 2 (n = 66)**			**P value**
	**Anti-HBc Positive (n = 20)**	**Anti-HBc Negative (n = 59)**	**P value**	**Anti-HBc Positive (n = 9)**	**Anti-HBC Negative (n = 57)**	**P value**	
Age, y, mean ± SD	46.6 ± 10.6	45.8 ± 10.3	0.7	47.3 ± 8.3	46.8 ± 10.3	0.8	0.9
Sex, No (%)			0.1			0.4	0.1
Male	7 (35)	35 (59.3)		4 (44.4)	36 (63.1)		
Female	13 (65)	24 (40.6)		5 (55.5)	21 (36.8)		
Duration of hemodialysis, mo, mean ± SD	35.7 ± 10.4	28.7 ± 10.7	0.05	32.2 ± 11	30.7 ± 10.07	0.6	0.03
Positive history of transfusion, No. (%)	20 (100)	50 (84.7)	0.02	9 (100)	46 (80.7)	0.07	0.01
Positive schistosoma antibodies, No. (%)	16 (80)	35 (59.3)	0.04	5 (55.5)	31 (54.3)	0.4	0.01
AST, IU/L, mean ± SD	29 ± 7	27 ± 13	0.4	28 ± 6	26 ± 12	0.4	0.6
ALT, IU/L, mean ± SD	30 ± 11	26 ± 18	0.3	30 ± 11	26 ± 18	0.5	0.7
Albumin, g/dL, mean ± SD	3.6 ± 0.4	3.7 ± 0.1	0.4	3.5 ± 0.5	3.6 ± 0.3	0.4	0.6
Bilirubin, mean ± SD	1.3 ± 0.5	1.2 ± 0.4	0.3	1.5 ± 0.6	1.4 ± 0.7	0.6	0.1
Anti-HBs (positive)	7 (35)	17 (28.8)	0.2	5 (55.5)	15 (26.3)	0.03	0.9
HBV DNA positive, No (%)	3 (15)	1 (1.6)	0.004	2 (22.2)	0	-	0.6

HBV DNA seropositivity was not associated with age, sex, history of hepatitis, history of blood transfusion, level of schistosomal antibodies, aminotransferase activity, or serum albumin. As expected, the presence of HBV DNA was significantly associated with anti-HBc and anti-HBe positivity (P = 0.003 and P = 0.002, respectively; Table 4). While more patients without HBV DNA more patients had anti-HBs, the correlation was not statistically significant (P = 0.1).

**Table 4:** Epidemiological, Biochemical, and Serological Markers of HBV in 145 Patients With ESRF Grouped by HBV DNA Status (Mean ± SD)

	**HBV DNA a Positive (n = 6)**	**HBV DNA Negative (n = 139)**	**P value**
Age, y, mean ± SD a	47.2 ± 10.6	46.8 ± 10.3	0.9
Sex, No. (%)			0.15
Male	2 (33.3)	(43.6)	
Female	4 (66.7)	(56.4)	
Duration of hemodialysis, mo, mean ± SD	34.2 ± 11.8	31.5 ± 10.4	0.5
Positive history of transfusion, No, (%)	6 (100)	119 (85.6)	0.1
Positive schistosoma antibodies	4 (66.6)	60 (43.1)	0.77
	2 (33.3)	79 (56.8)	
AST a , IU/L, mean ± SD	27 ± 8	22 ± 19	0.5
ALT a , IU/L, mean ± SD	28 ± 11	26 ± 20	0.8
Albumin, g/dL, mean ± SD	3.46 ± 0.5	3.5 ± 0.6	0.8
Bilirubin, mean ± SD	1.4 ± 0.6	1.3 ± 0.5	0.6
Anti-HBc a positive, No. (%)	5 (83.3)	24 (17.2)	0.003
Anti-HBs positive, No. (%)	1 (16.6)	43 (30.9)	0.1

^a^ Abbreviations: ALT, alanine transaminase; Anti-HBc, hepatitis B core antibody; AST, aspartate transaminase; HBV DNA, hepatitis B virus DNA by polymerase chain reaction; SD, standard deviation

## 5. Discussion

The present study enrolled HBsAg negative patients, regardless their HCV RNA status, and demonstrated that 4.1% of Egyptian patients with ESRF from Upper Egypt had OBI. This prevalence did not differ significantly between patients with and without HCV RNA (i.e., 5.06 vs. 3.03 in Group 1 and Group 2, respectively). Previous reports from studies in dialysis units have indicated that the prevalence of OBI ranges from 0% to 58% among patients [2][20][21][22]. This wide range of estimates may attributable to differences in the sensitivity of the methods used for the detection of HBV DNA [23], the patients’ sample investigated in each study, and the level of HBV endemicity in the populations of the different geographic areas. The most accurate method for OBI testing is the analysis of liver DNA extracts and the detection of HBV DNA in serum samples may underestimates the true prevalence of OBI [24][25]. However, the performance of liver biopsies in patients undergoing HD is often very difficult, and is usually contraindicated. A similar prevalence of markers of anti-HBc and HBV DNA has recently been found among Egyptian blood donations [26].

In this study, HBV DNA seropositivity was not associated with age, sex, history of hepatitis, history of blood transfusions, levels of schistosomal antibodies, aminotransferase activity, or serum albumin. However, there were significant associations between the presence of HBV DNA and anti-HBc positivity, and between HBV DNA and anti-HBe positivity. The absence of HBV DNA was associated with the presence of anti-HBs. The prevalence of anti-HBc was 20%. As anti-HBc positivity indicates previous exposure to HBV infection, this finding in our HD patients in association with negative HBsAg might suggest contact with HBV sometime during adulthood. However, this cannot be safely assumed for HD patients. Although anti-HBc levels were higher in Group 1 (21.9%) than in Group 2 (11.5%), this difference was not statistically significant. This ratios are in consistent with the available data in the literature [2][21][27][28].

OBI may have an impact in different clinical contexts, including possible transmission of the infection [24][25], risk of reactivation [24][25], contribution to liver disease progression [29], and development of hepatocellular carcinoma [30]. Clinical interactions between HCV and OBI are still controversial. Studies performed in different geographic areas have shown a significant association between OBI and severe liver diseases, particularly in patients with HCV infection. This suggests that OBI may favor or accelerate the progression toward cirrhosis in these individuals [31]. Mild necroinflammation can be revealed many years after the resolution of acute hepatitis Infections. This may explain how a persistent, yet suppressed, viral infection may have pathogenic activity. As they have similar transmission modes, HBV and HCV co-infection are common clinical presentations. Because prevention is the first line of defense against viral hepatitis infection and is much more cost-effective than treatment, prevention should be the main goal of current efforts to break the vicious cycle of the infection.

In Egypt, HBV vaccination of newborns has been available for populations at high risk of HBV infection since 1992. However, neither children nor young adults were provided with “catch-up vaccinations” [32]. The government should provide more support for the vaccine, especially for the vaccination of high-risk groups, such as HD patients and medical staff, as the cost of treatment is much higher than the cost of vaccination. The prevalence of OBI among high-risk groups, such as HD patients, can only be definitively determined from a large, national epidemiological study. As there is no such national sample survey in Egypt, our study provides a useful compromise. This study demonstrated that greater attention on the part of government health authorities is still needed for appropriate prevention of viral hepatitis. Our study findings highlight the significance of continuing prevention of HBV through vaccination campaigns as well as the development of an integrated strategy for primary prevention that focuses on identification of the risk factors that may, if addressed, help curtail the spread of HBV infections in developing countries.
